# Unusual to “Bee” in the Colon: A Rare Finding on Screening Colonoscopy

**DOI:** 10.14309/crj.0000000000001260

**Published:** 2024-02-07

**Authors:** Muhammad Nadeem Yousaf, Matthew L. Bechtold

**Affiliations:** 1 Division of Gastroenterology and Hepatology, Department of Medicine, University of Missouri, Columbia, MO; 2 Gastroenterology, Harry S. Truman Veterans Administration, Columbia, MO

## CASE REPORT

**Figure 1.**
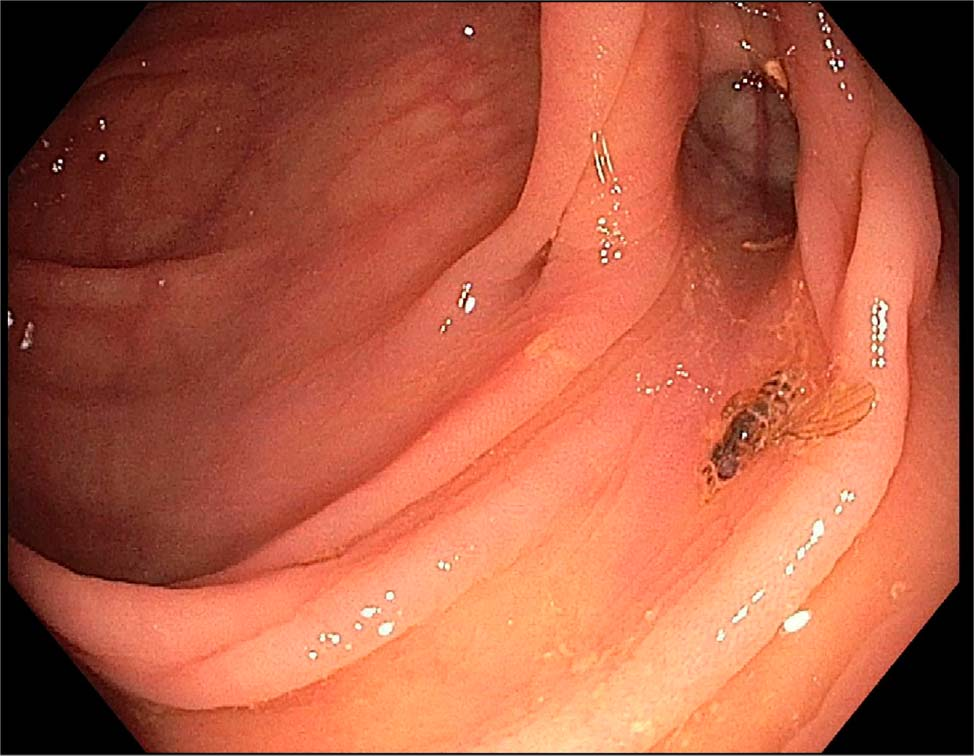
A honey bee in the decending colon.

**Figure 2.**
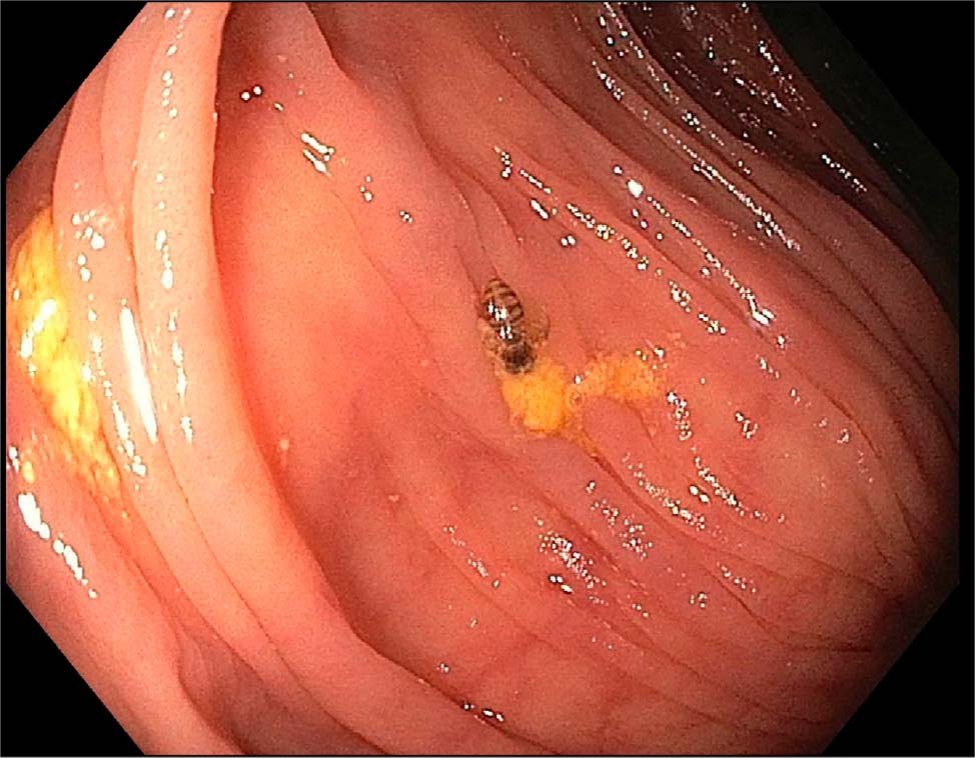
A honey bee in the decending colon.

**Figure 3.**
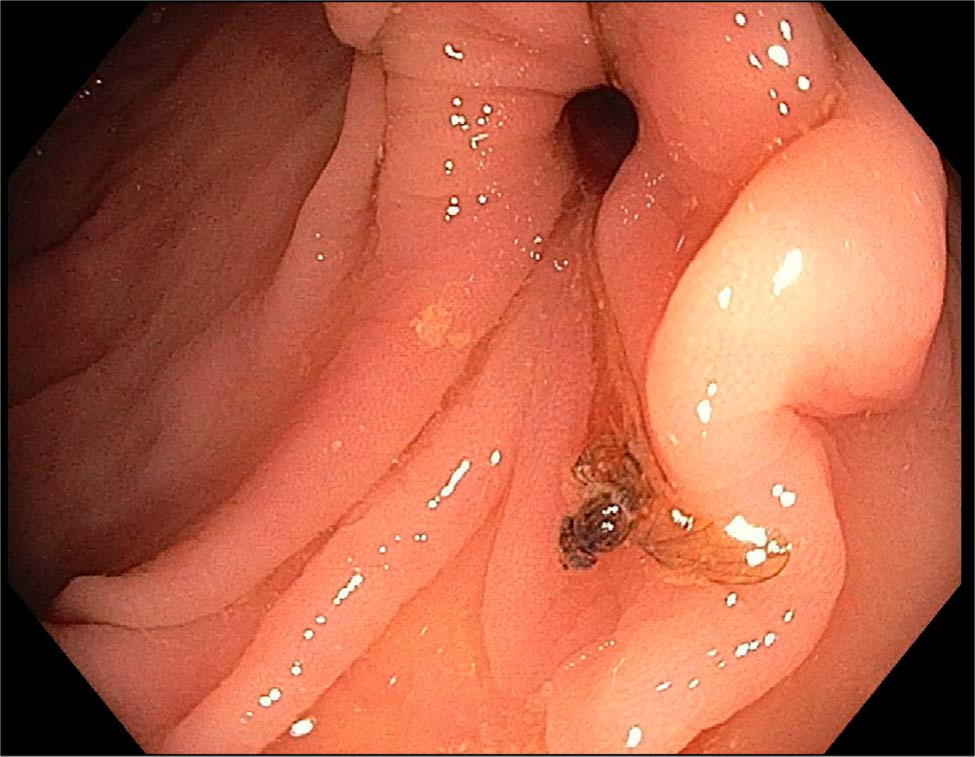
A honey bee in the decending colon.

A 78-year-old man with a medical history of esophageal cancer status post esophagectomy 5 years ago and family history of colon cancer in a sister was referred by his primary care provider for screening colonoscopy. The patient had split-dose bowel preparation with 4 L of polyethylene glycol with electrolytes. During colonoscopy, he was noted to have a 7 mm inflammatory (Paris 1S) polyp in the cecum, which was removed with a cold snare, and pancolonic diverticulosis. In the descending colon, a honey bee was noted that was removed with suction (Figures 1–3). The patient stated that the day before colonoscopy, he was biking and ate grapes before starting bowel preparation. He believed he swallowed the honey bee while biking. Honey bee stings contain venom that may cause significant pain, allergic reaction, and anaphylaxis reaction in severe cases.^1^ A honey bee in the colon during colonoscopy is a very rare finding. It is interesting that an intact swallowed honey bee was found in the descending colon despite upper GI enzymes, clear liquid diet, and bowel preparation.

## DISCLOSURES

Author contributions: MN Yousaf: patient care, manuscript writing. ML Bechtold: proofread and edits of the manuscript, and supervised in finalizing the manuscript. ML Bechtold is the article guarantor.

Financial disclosure: None to report.

Informed consent was obtained for this image case report.
